# Potential miscommunication of risk in American College of Radiology Prostate Imaging and Data System (PIRADS) scores

**DOI:** 10.1038/s41391-023-00781-0

**Published:** 2024-01-03

**Authors:** Jeremiah R. Dallmer, Brennan Spiegel, Stephen J. Freedland, Rola Saouaf, Paige Kuhlmann, Timothy J. Daskivich

**Affiliations:** 1 Department of Urology, Cedars-Sinai Medical Center, Los Angeles, CA USA; 2 Cedars-Sinai Center for Outcomes Research and Education (CS-CORE), Cedars-Sinai Medical Center, Los Angeles, CA USA; 3 Department of Medicine, Divisions of Gastroenterology and Health Services Research, Cedars-Sinai Medical Center, Los Angeles, CA USA; 4 Section of Urology, Durham VA Medical Center, Durham, NC USA; 5 Department of Imaging, Cedars Sinai Medical Center, Los Angeles, CA USA

**Keywords:** Prostate cancer, Prostate cancer

In May of 2020, the United States Office of the National Coordinator for Health Information Technology Final Rule implemented sections of the 2016 21st Century Cures Act, which ensures that patients in the USA have direct access to their health records, including physicians’ notes, lab values, and diagnostic reports [1]. Though the electronic medical record (EMR) has traditionally been a provider-focused data repository intended for treatment, billing, and legal documentation, the ONC Cures Act has expanded its purpose to include patient engagement and education. While the Cures Act is laudable in its pursuit of transparency, direct access to the electronic medical record (EMR) also confers the potential for patient misunderstanding given a lack of clinical knowledge and context. As such, the use of precise, patient-focused language in medical records has become more important to ensure clarity and avoid unnecessary confusion among patients, especially for high-risk, emotionally evocative conditions such as cancer.

Standardized language endorsed by the American College of Radiology (ACR) to describe prostate MRI results provides an example of how imprecise language in the EMR can lead to confusion, anxiety, and even a false presumption of cancer diagnosis by patients. The goal of diagnostic prostate MRI is to identify areas of concern for clinically significant cancer that can be targeted on biopsy to confirm or deny presence of malignancy. Prostate cancer is generally considered clinically significant when involving Gleason 3 + 4 disease (grade group 2) or higher [2, 3]. Interpretation of prostate MRI has been standardized by the ACR under the Prostate Imaging and Reporting and Data System Version 2.1 (PIRADS-V2.1), with scoring ranging from 1-5 based on information from diffusion-weighted imaging, apparent-diffusion coefficient, T2, and contrast enhanced perfusion multiparametric MRI sequences, with a higher score indicating a higher probability of prostate cancer. The ACR also endorses standardized descriptions of the likelihood of each score in identifying clinically significant cancer as noted in Table 1, ranging from “highly unlikely” (PIRADS 1), “unlikely” (PIRADS 2), “equivocal” (PIRADS 3), “likely” (PIRADS 4), to “highly likely” (PIRADS 5). By definition, the term “likely” implies a probability >50%, and the term “equivocal” implies uncertainty or ambiguity (i.e. 50/50 probability). However, the diagnostic characteristics for detection of clinically significant prostate cancer by PIRADS scores of 3 and 4 are out of step with these definitions. Guidelines from the American Urologic Association cite the rate of detecting clinically significant prostate cancer for PIRADS 3 and 4 lesions as 11% and 37%, respectively [4]. Therefore, by ACR standard descriptions, an 11% probability for PIRADS 3 lesions corresponds to an “equivocal” likelihood, and a 37% probability for PIRADS 4 lesions corresponds to a “likely” diagnosis of clinically significant prostate cancer. This loose language can result in inappropriate assumptions about a cancer diagnosis by patients reading the EMR, as illustrated by the following quotes from patients in our practice who had PIRADS 4 lesions and ultimately went on to have negative biopsies:

*“I just received the MRI on the link. I see it’s saying I might have cancer…Please contact me as soon as possible.”*

*“I hope by now you have had an opportunity to review my recent MRI. Obviously, I am somewhat concerned by the results…”*

**Table 1:** PI-RADS scores and interpretation according to the American College of Radiology [3].

PI-RADS v2.1 Assessment Categories
PIRADS 1—Very low (clinically significant cancer is highly unlikely to be present)
PIRADS 2—Low (clinically significant cancer is unlikely to be present)
PIRADS 3—Intermediate (the presence of clinically significant cancer is equivocal)
PIRADS 4—High (clinically significant cancer is likely to be present)
PIRADS 5—Very high (clinically significant cancer is highly likely to be present)

Inappropriate attribution of a cancer diagnosis to a patient is unacceptable, since even a brief misunderstanding may cause significant anxiety for the patient and their family. While physicians could preemptively discuss the use of MRI in risk assessment prior to imaging, the language in the report would still be misleading. Receiving a radiology report which describes an MRI finding as “equivocal” or “likely” to represent clinically significant cancer for PIRADS 3 and 4 lesions that have 11 and 37% likelihood of truly being clinically significant, respectively, is without question misleading. After implementation of the Cures Act, when patients will often receive this information outside of a conversation with their physician, standard language of the PI-RADS scoring system (Table 1) should be revised and/or be accompanied by actual estimates (ideally institution- or radiologist-specific estimates, given high variability between reading radiologists) to better reflect true risks of disease. This is consistent with how other cancer screening tests for prostate cancer (e.g. PSA and free PSA) are reported and how other standardized imaging reports such as the Breast Imaging Reporting and Data System are presented [5]. It is time for the ACR to update PI-RADS language to be more consistent with actual risk.
